# Correlates of male fitness in captive zebra finches - a comparison of methods to disentangle genetic and environmental effects

**DOI:** 10.1186/1471-2148-11-327

**Published:** 2011-11-08

**Authors:** Elisabeth Bolund, Holger Schielzeth, Wolfgang Forstmeier

**Affiliations:** 1 Department of Behavioural Ecology & Evolutionary Genetics, Max Planck Institute for Ornithology, Eberhard-Gwinner-Strasse 5, Seewiesen, 82 319, Germany; 2 Department of Animal and Plant Sciences, University of Sheffield, Sheffield, S10 2TN, UK; 3 Evolutionary Biology Centre, Uppsala University, Norbyvägen 18D, Uppsala, 752 36, Sweden

## Abstract

**Backgound:**

It is a common observation in evolutionary studies that larger, more ornamented or earlier breeding individuals have higher fitness, but that body size, ornamentation or breeding time does not change despite of sometimes substantial heritability for these traits. A possible explanation for this is that these traits do not causally affect fitness, but rather happen to be indirectly correlated with fitness via unmeasured non-heritable aspects of condition (e.g. undernourished offspring grow small and have low fitness as adults due to poor health). Whether this explanation applies to a specific case can be examined by decomposing the covariance between trait and fitness into its genetic and environmental components using pedigree-based animal models. We here examine different methods of doing this for a captive zebra finch population where male fitness was measured in communal aviaries in relation to three phenotypic traits (tarsus length, beak colour and song rate).

**Results:**

Our case study illustrates how methods that regress fitness over breeding values for phenotypic traits yield biased estimates as well as anti-conservative standard errors. Hence, it is necessary to estimate the genetic and environmental covariances between trait and fitness directly from a bivariate model. This method, however, is very demanding in terms of sample sizes. In our study parameter estimates of selection gradients for tarsus were consistent with the hypothesis of environmentally induced bias (*β*_A _= 0.035 ± 0.25 (SE), *β*_E _= 0.57 ± 0.28 (SE)), yet this differences between genetic and environmental selection gradients falls short of statistical significance.

**Conclusions:**

To examine the generality of the idea that phenotypic selection gradients for certain traits (like size) are consistently upwardly biased by environmental covariance a meta-analysis across study systems will be needed.

## Background

**Figure 1:**
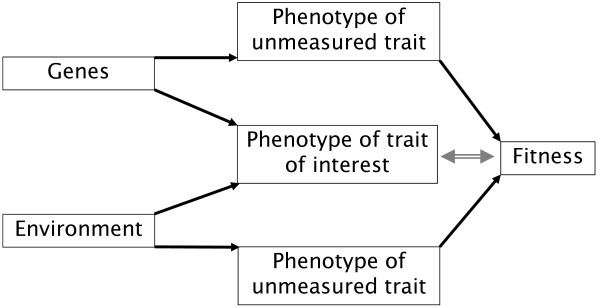
**A correlation (grey, double-sided arrow) between the phenotypic expression of a trait and a measure of fitness is often assumed to be a causal relationship, i.e. the phenotype is thought to affect fitness**. Therefore, the genetic and environmental correlations of the trait with fitness are expected to be the same as the phenotypic correlations with fitness (r_A _= r_E _= r_P_). However, correlation does not imply causation, and the causal route (black single headed arrows) may go via one or more unmeasured phenotypic traits that may be correlated with the trait of interest. In this scenario, phenotypic selection patterns need not be the same as genetic selection patterns, and a genetic perspective is necessary to elucidate patterns of response to selection.

Selection acts on the phenotypes of individuals, while an evolutionary response to selection requires genetic transmission to following generations [1]. Thus, only if the genetic component of the trait is related to fitness it will lead to an evolutionary response [2], a condition that is likely to be fulfilled if the trait is causally affecting fitness. If only the non-heritable environmental component of the trait is related to fitness, there will be no evolutionary response [3,4]. This is likely to be the case if the trait is not causally related to fitness, but is correlated to a fitness-related trait via environmental condition dependence. For example, one might observe a positive correlation between a phenotypic trait like body size and fitness in a population of birds (Figure 1). It may be that a large body size reflects good early growth conditions and that these good conditions constitute the causal link to fitness (by affecting other aspects of the phenotype e.g. health). Thus, in this example a large body size per se does not cause higher fitness, and the environmental components of body size are not inherited to the next generation. Therefore, despite apparent selection on body size, there would be no response to selection, and body size would remain stable over evolutionary time. The existence of such an environmental bias in the estimates of selection could explain an important phenomenon in evolutionary biology. Numerous studies on wild animal populations have found consistent positive selection for body size, condition or laying date without finding any indication of a resulting evolutionary change [reviewed in [5]]. Attempts to resolve this problem have invoked such an environmental bias and tried to decompose the phenotypic selection into its genetic and environmental components. However, these studies have often still found significant selection at the genetic level despite a lack of evolutionary response [e.g. [6-8]]. The key to resolving this issue may lie in the methodology employed to decompose the phenotypic selection and we will therefore briefly outline the history of the methods used below.

Information about selection on the genetic level is essential to fully understand evolutionary processes. The importance of a genetic perspective has been pointed out repeatedly [e.g. [9,10] ], yet focus often remains on the phenotypic level. This is due to two main reasons. First, the assumption that phenotypic selection patterns will largely reflect genetic selection patterns (Figure 1, termed the 'phenotypic gambit' by [11]). This assumption was supported by studies comparing genetic (r_A_) and phenotypic (r_P_) correlations with fitness [e.g. [12,13] ]. Importantly, the support came mainly from studies on traits with high heritabilities, i.e. where the genetic component makes up a large portion of the total phenotypic variation (in this situation r_P _is likely to be similar to r_A_). Second, a genetic perspective places high demands on the data used since it requires extensive pedigree information and large sample sizes that are not easily feasible in most study populations. Further, the statistical analyses are computationally demanding and rely on methods that have been developed in other fields of research [14].

Nevertheless, there has been a surge of studies looking at the strength and direction of selection and evolutionary response to selection [see [14-18] ]. As alluded to above, this has been made possible by the application of quantitative genetic methods that use pedigree information to decompose trait variation into the underlying genetic and environmental variances. These methods have been used for more than 50 years in animal breeding, where identification of genetically promising individuals is of great interest for the artificial selection on relevant traits, but have only relatively recently entered the field of evolutionary ecology [reviewed in [19] ].

The history in animal breeding has had an important influence on the way that selection is estimated. In animal breeding, the focus is on identifying genetically promising individuals in order to induce responses in specific traits (e.g. milk yield in cattle). This has lead to a methodology favouring the estimation of individual level parameters, namely the genetic merit of the individual. The genetic merit of an individual cannot be measured directly, but it is possible to obtain an estimate of the expected effect of the genes that the individual passes on to its offspring. This is called the predicted breeding value (BV) of the individual [14,20]. An individual's BV is twice the expected deviation of its progeny from the population mean, when the individual is mated randomly to other individuals from the population [20]. Animal breeders use breeding values to impose selection by choosing animals with high or low BVs. In contrast, an important goal in evolutionary studies is to infer selection patterns from the BVs across generations. That these differences in the focus of study might warrant different methodologies has not been fully appreciated [see [21]]. Recently, the suitability of using breeding values to estimate selection patterns has been severely criticized [22-24]. We will briefly review the reasons for this caution against the use of breeding values and the alternatives that have been suggested. For a more detailed discussion [see [22-24]].

Selection pressures can be quantified by selection gradients *β*, i.e. the regression slope of relative fitness on trait values [2,20]. The response to selection R can be predicted by the product of selection gradients and the additive genetic variance R = β*V_A _[e.g. [25], see [26] for an excellent critical review regarding the use of the breeder's equation]. This relies on the assumption that selection at the genetic level of the traits *β*_A _has the same strength as selection at the phenotypic level *β*_P _(the 'phenotypic gambit'). This condition is likely to be fulfilled if the trait is causally relevant for fitness, but not necessarily if the trait is merely correlated to some fitness-relevant trait. Rausher [4] suggested that the selection could be better calculated at the genetic level. This can better inform us if the phenotypic selection will result in evolutionary change.

In the case of a single trait, the genetic selection gradient (*β*_A_) is defined as the genetic covariance between the trait and relative fitness (σ_a,w_) divided by the additive genetic variance in the trait (σ_a_^2^) [27]:

(Eq.1) $$\beta_{\text{A}}=\frac{\sigma_{a,\omega }}{\sigma_{a}^{2}}$$

The selection analysis can be easily extended to the multivariate case, to account for selection on correlated characters [28]. We will here for simplicity focus on the univariate case. The breeding value approach alluded to above entails first estimating the additive genetic effect for each individual (estimating а using estimated breeding values, â) and then regressing individual fitness on this measure. Best linear unbiased predictors (BLUPs) of breeding values can be estimated with the use of pedigree data (the so-called 'animal model', [29]). Thus, the phenotypic value of the individual itself and the values of all its relatives are weighted by the degree of relatedness and used to estimate the genetic component of the individual. However, BLUPs (predicted BVs) are point estimates of the true BVs, and the variance in predicted BVs is always lower than the variance in true BVs [23,24].

To determine (on the population level) how close the predicted BVs are to the true (but unknown) BVs, we can calculate the reliability of the predicted BVs. This gives the proportion of the additive genetic variance (or the variation in true BVs) that is explained by the predicted BVs [24,30]. The reliability can be calculated by dividing the variance in the predicted BVs with the additive genetic variance [30,31]. Typically, reliability estimates are around 50% in studies on wild populations [see [31]]. One might assume that this unreliability of predicted BVs would make estimates of genetic correlations more conservative, in the sense of biasing them towards zero. However, the statistical model assumes that the genetic correlations are the same as the phenotypic correlations [24]. Therefore, the unreliability will result in a bias towards the phenotypic correlation when genetic correlations are estimated in a two-stage analysis by correlating BVs with fitness [23].

Another reason that will bias estimates of genetic selection gradients based on predicted BVs is that BV estimation is based not only on the phenotypes of relatives but also the phenotype of the focal individual. Thus, the estimated BV will include some amount of environmental variation in addition to the genetic variation, and will thus partly reflect the environmental component of the individual's phenotype [24]. This problem becomes more pronounced as the heritability of the trait decreases, because the phenotype of an individual will to a larger extent be determined by environmental variation, while at the same time, relatives do not contribute much independent information. The problem is also exacerbated by small sample sizes (e.g. poorly linked pedigrees or traits with a sex limited expression), because there are fewer relatives that can contribute to BV estimation. As the amount of information available from relatives declines, the influence of an individual's own phenotype for estimating its BV increases. Postma *et al. *[24], suggested an alternative approach of calculating BVs to remedy this problem. The phenotype of a target individual can be removed from the data file when calculating its BV. The BV, calculated only from the phenotypes of relatives, will be more or less free from environmental variation (to the extent to which there are no shared environmental effects among relatives). Such calculations can be done iteratively for all individuals in the population. However, since this relies on perfect additive inheritance and ignores chance effects during segregation, a small part of the additive genetic variance will be systematically assigned to the residual variation. Furthermore, because the information on the individual phenotype is discarded, less information is available to predict the BV for each individual, which will result in more uncertainty around individual BVs. Therefore, while BVs calculated without individual phenotypes will not systematically include parts of the environmental component of the individual's phenotype, the reliability of these BVs will be lower and this might cause problems in further analyses.

A number of problems with the approach of first estimating BVs and then relating them to fitness was recently pointed out by Hadfield [[22], see also [19]]. Since BVs are estimated in a model without fitness (fitness is treated by the model as a missing trait), the resulting genetic selection gradient will be strongly biased towards the phenotypic selection gradient. To resolve this problem, we can fit a bivariate model of fitness and the trait and calculate genetic selection gradients directly from the estimated genetic variance-covariance matrix **G **(estimating σ_a,ω _and σ_a_^2 ^simultaneously), thus by-passing the need for individual BVs [see [22,23]]. A limitation to this method arises if the additive genetic variance for fitness is low, since this will require very large sample sizes to estimate the covariance between trait and fitness (and hence the genetic selection gradient). This method of calculating genetic selection gradients has yet to be widely appreciated in evolutionary biology, although a few studies have reported genetic correlations between trait and fitness [e.g. [32,33]].

The first aim of the present study is to compare the three different methods of estimating genetic selection gradients that have been outlined above. We (1) calculate predicted BVs using the full phenotypic information on all individuals and (2) calculate BVs with the phenotype of the individual in question removed from the data file when calculating its BV. Under (1) and (2), The BVs are calculated using an animal model with one trait only and are then related to fitness with a regression approach. Finally (3), we estimate genetic selection gradients from the genetic covariances between trait and fitness directly in a two-trait model that includes both the trait of interest and fitness, following Hadfield [22].

We use a model species in studies of sexual selection, the zebra finch, to look at patterns of selection. The zebra finch is socially monogamous with some extra-pair paternity [34]. A plethora of studies have focused on female preferences for male beak colour and courtship song rate (traditionally termed 'directed song' rate in estrildids, [34], henceforth 'song rate'), either in choice chamber type set-ups (reviewed in [35,36] or, in a few studies, under more realistic aviary conditions [37-41]). A majority of the studies have found or inferred a preference for redder beaks or higher song rates. However, no study so far has used genetic paternity assignment to relate male traits to success at gaining paternity, neither has any study decomposed the phenotypic selection into its genetic and environmental components. Looking at actual paternity patterns is especially important given previous findings from our population, since we have found no female preference for males with redder beaks in choice chamber trials [36] or for males with higher song rates in extra-pair copulation trials [42]. Previous evidence from our population showed that body size (measured as tarsus length) is of some importance in aggressive interactions [43], suggesting that it could be important for male success under aviary conditions. Thus, the second aim of the current study is to relate song rate, beak colour and tarsus length to an important fitness component: success at fertilizing eggs under free-flying aviary conditions, and decompose the selection acting at the phenotypic level into genetic and environmental selection gradients to see if the observed selection can lead to an evolutionary response.

It might be argued that the investigation of selection pressures in captivity has limited validity and cannot be generalized to the situation in the wild. However, it is important to note that evolution continues after taking birds into captivity, albeit under changed selection pressures. Therefore, while selection pressures estimated in captivity may not reflect what is happing in the wild [but see e.g. [44]], the findings can nevertheless be informative about evolutionary processes in general. Importantly, the captive situation allows us to obtain high quality pedigree information, a separation of genetic and environmental effects by means of cross-fostering and accurate measurements under standardised conditions of the three target traits. This provides the basis for fairly accurate breeding value estimation. We also obtain very good data on a fitness component from a subset of birds that were allowed to breed for two breeding seasons in aviaries. Captive breeding allows the exclusion of stochastic variation in fitness due to e.g. largely random nest predation and the opportunity to obtain perfect knowledge of paternity for a large number of eggs. However, the labour-intensive measurement of fitness limits our data on fertilisation success to 68 males. This moderate sample size will still lead to relatively large standard errors of the estimates, but will not lead to any consistent bias, if the tested subset is representative of the whole population. The estimated selection gradients with this sample size should be interpreted with caution, and serves rather as a proof of principle than as precise estimates of selection pressures in captive zebra finches.

## Methods

### Subjects and housing

All subjects were kept at the Max Planck Institute for Ornithology in Seewiesen, Germany, since October 2004. For details of rearing conditions for these birds, see [45]. Rooms were maintained at a constant temperature of 24 ± 1°C, with humidity ranging from 40 to 60%. Birds received a millet seed mixture, cuttlefish, grit and water ad libitum. The diet was supplemented once a week with salad and a multivitamin supplement. Rooms were illuminated by full spectrum fluorescent light (Osram Lumilux T5 FH 28W/860 Daylight) on a 14:10 h light:dark photoperiod. For details of the breeding set-up, see [46].

### Measurements of traits

To measure directed song rate, we performed 5-minute male-female encounters involving socially unpaired males and females. To maximise statistical power, we included both pair-wise encounters [47] and encounters in trios [43]. In each trial we measured the total duration (in seconds) of male song directed towards the female and labelled this the directed song rate of an individual. Following [48], the measurements were square-root transformed to approach normality. Song rate was measured repeatedly over the life-time in this way (in separate 'trial batches'), males participated in on average 7.16; SD 3.93, trials (N = 586 males). The subset of males that participated in the current breeding experiment had an average of 12.8; SD 1.07 song measures. Each male received a life-time score of directed song rate by taking the random effect estimates (BLUPs) of song rate from a linear mixed effect model, controlling for trial batch, cohort effects and whether birds were reared in unisexual or mixed sex groups (all focal males were raised in unisexual groups) as fixed effects. BLUPs of song rate had a mean of 0 and SD of 1.50 seconds^1/2^.

Beak colour was measured with spectrophotometry to capture the variation over the full bird visible spectrum (320-700 nm), including the UV-part. Beak colour was measured at several occasions over the life time for all birds (mean 1.8; SD 1.1, measurements, N = 1019 birds), always under nonbreeding conditions (because beak colour changes dramatically during the breeding season [49] and mate choice takes place before breeding is initiated). The subset of males that participated in the present experiment were measured at around 100 days of age, at the start of each of the two breeding rounds in the aviaries, and, for a subset of 32 males, also at two later non-breeding occasions. From the beak colour spectrograms, we extracted 6 spectral characteristics that describe the shape of the spectral curve and captures the points of maximal sexual dimporhism [43], which were corrected for measuring batch and sex of the bird as fixed effects and precise age at measure as a continuous fixed effect, by taking random effect estimates from linear mixed effect models. Sex was added as a fixed effect to account for the pronounced sexual dimorphism in beak colour. Male and female beak colour is strongly genetically correlated (r_A _= 0.93, CI: 0.67-1.06 [50], see also [51]: r_A _= 0.91 ± 0.12 (SE)). Thus, removing the sex effect allowed us to include female beak colour when running quantitative genetic analyses to improve the estimates of breeding values of beak colour. We then extracted a PC1 of the 6 spectral characteristics as described in Bolund *et al*. ([43], loadings were very similar to the values reported therein). The PC1 ranges from -0.96 to +0.86 (mean 0; SD 0.27) and mainly captures beak colour variation on a female to male axis, with lower scores reflecting beaks that are more orange (as opposed to red) and have a higher reflectance peak in the UV.

Tarsus length was measured to the nearest 0.1 mm at 35-367 days of age (mean = 1.44 measures, range 1-2, N = 1049 birds). For birds that were measured twice, we used the average of the two measures (the tarsus does not change in length after 35 days of age). Tarsus length showed a mean of 17.2; SD 0.59, mm.

For song rate and beak colour, we chose to use BLUPs as a means of averaging across unequal sample sizes while controlling for fixed effects. This allows maximal simplification of the animal models which was highly desirable to ease the comparison of the different analytical approaches (see below). Two alternatives would be to (1) Enter all measurements of each individual into the animal model and account for repeated measures by adding a permanent environment effect or (2) Use simple averages or only one measurement per individual. Both these alternatives require fixed effects to be added to the animal model (trial batch, cohort effects and unisex versus mixed sexed rearing in the case of song rate) that are a nuisance to the estimation of the covariance between trait and fitness. To test the validity of our chosen approach, we tested these alternative approaches in MCMCglmm and found that alternatives 1 and 2 sometimes lead to problems with convergence of the animal models, possibly due to power issues as the sample size for fitness was very limited. In cases where all three approaches could be compared results did not differ qualitatively (not shown) and conclusions remain the same.

### Aviary breeding

Six females and six males in each of nine large hexagonal free-flight aviaries were allowed to breed for three months in September-November 2005 (birds were on average 484; SD 29, days old at the start of the experiment). Birds were chosen from the third generation in a five-generation pedigree. When choosing birds for the aviary experiment, we ensured that all families were represented and that no bird shared an aviary with a relative or a bird it had at any point been housed together with. As part of a sex-ratio treatment, three aviaries had an additional three females, three aviaries an additional three males, while the remaining three had no additional birds added, giving sex ratios of 0.4, 0.6 and 0.5, respectively [52]. After a non-breeding period without nesting opportunities, breeding pairs were exchanged among aviaries and sex-ratio treatments, such that each pair faced five new unfamiliar pairs, and allowed to breed for another three months in April-June 2006. Thus, most males bred with the same partner in two different sex ratio conditions. In total, due to the replacement of dead birds, 71 females and 68 males were used. The paternity of eggs or offspring was determined using 10 microsatellite loci [53], and assigned to parents by exclusion. Out of all fertile eggs, 96.3% were sampled and 99.9% of these were assigned unambiguously to a genetic father and a genetic mother (for details of the paternity assignment, see [46]). Calculations are based on 1727 eggs. For each male, we calculated the overall number of eggs fertilised, which is an important fitness component. This includes eggs laid by the partner, by extra-pair females and by unpaired females. We calculated the repeatability of male relative success (number of eggs a male fertilised divided by the mean number of eggs fertilised within the aviary) from a linear mixed effect model. The ratio of the identity variance component to the total variance is the intra-class correlation and represents the individual repeatability [54]. Repeatability was 0.65 (LRT: χ^2^_1 _= 34.1, P < 0.0001) for overall fertilisation success.

### Quantitative genetics

#### Breeding value estimation

We used animal models fitted using REML-VCE 6.0.2 [55] to estimate how much of the phenotypic variance in tarsus, song rate and beak colour is due to additive genetic, maternal and early environmental effects. To estimate additive genetic components we used a 5-generation pedigree with N = 1374 birds and perfect knowledge of the parentage of all individuals [46,56]. Of the generations with phenotypic data, 72% of the birds in generation two, 99% of generation three, 100% of generation four and 0% of the birds in generation 5 were cross-fostered. In total, 82% of birds with phenotypic data were cross-fostered.

The 68 males in the breeding experiment had an average of 15.8; SD 7.3, close (r = 0.5) relatives in the pedigree and an average of 25.8; SD 17.3 relatives with r = 0.25 (counting only relatives with phenotypic data for tarsus available), which should allow fairly accurate estimation of breeding values. The average level of inbreeding in this laboratory population is relatively low (F = 0.03, based on an 18-generation pedigree, [48], close inbreeding has been consistently avoided). Phenotypic data on tarsus, beak colour and song rate were available for all except the first generation of the pedigree, N_tarsus _= 1054 males and females, N_beak colour _= 1019 males and females, N_song rate _= 586 males. Mother identity was entered to estimate maternal effects (additional to the additive genetic contribution of the mother, N_tarsus _= 251 mothers, N_beak colour _= 212, N_song rate _= 203), foster parent identity was entered to estimate rearing environment effects (N_tarsus _= 350 foster families, N_beak colour _= 303, N_song rate _= 279). For song rate and beak colour, which continue to change after day 35, peer group identity (i.e. the group the bird was kept in from independence at day 35 until full maturity at around day 100) was entered to estimate peer group effects (N_beak colour _= 82 peer groups, N_song rate _= 56).

**Figure 2:**
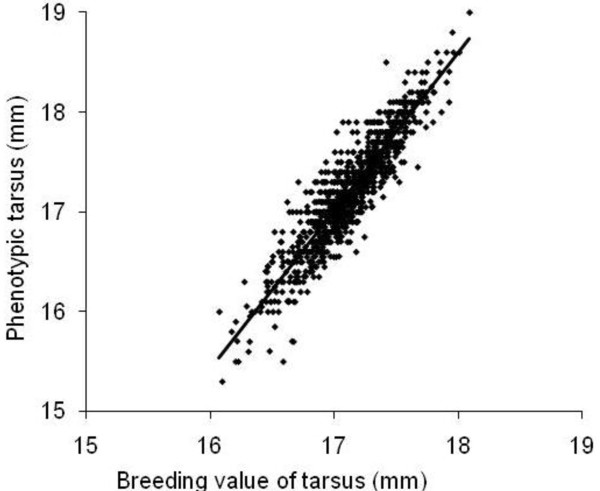
**Regression of the phenotypic value of tarsus on the breeding value (here BVincl) of tarsus**. The breeding value is expressed in mm by adding the population mean tarsus length to the BV of each individual. Thus, the x-axis illustrates the estimated genetically determined tarsus length that would be achieved under a standard environmental condition, while the y-axis illustrates the actual phenotypic tarsus length that was realised under the actual environmental conditions of the individual. The residuals from the regression represent the environmental contribution (RESincl) to tarsus for each individual. Note that estimates of BV are biased towards the phenotype because the phenotype of an individual contributes to the estimation of its BV (BVincl). Hence, the correlation is stronger (r^2 ^= 0.83) than expected from the heritability (h^2 ^= 0.55). The slope of the regression is 1.59.

To estimate the breeding value (BV) for each trait, we used the PEST software [57]. This program uses the REML estimates of variance components to estimate best linear unbiased predictors (BLUPs) of the breeding value. To obtain an estimate of the environmental component of each trait, the phenotypic value was regressed on the BV [24]. The residuals from this regression represent the environmental component of the trait (Figure 2).

**Figure 3:**
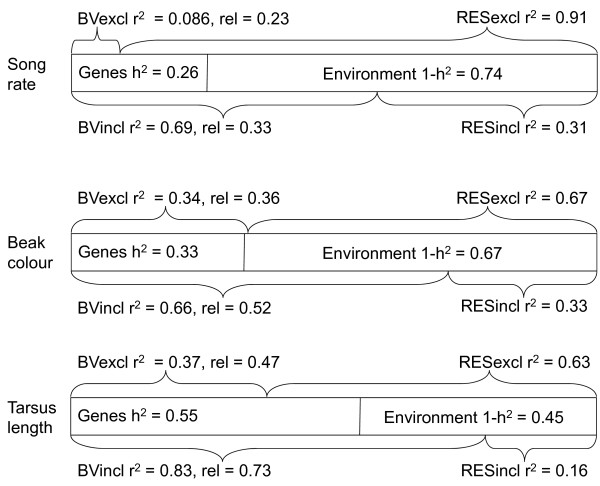
**The animal model approach divides the variation of a trait into a genetic (breeding value, BV) and an environmental (residual) component**. However, if the BV of a focal individual is calculated with the knowledge of the individual's phenotype, this BV (BVincl) will contain some part of the individual specific environmental component in addition to the genetic component. This can be remedied by excluding the focal individual's phenotype from the data file while calculating its breeding value (BVexcl). However, due to the loss of information the BV then reflects only part of the genetic variation. The figure illustrates how the genetic and environmental components of the three traits (song rate, beak colour and tarsus length) are represented by the two types of BVs and their corresponding residuals (RESincl and RESexcl, respectively). r^2 ^values refer to the amount of the total phenotypic variance that is explained by the BV or RES in question. 'rel.' refers to the reliability (see main text) of the estimated BVs. Note that due to the unreliability of the estimation of BVs even the RESincl will reflect some additive genetic effects and both the BVincl and BVexcl will not capture the entire additive genetic component and will also reflect some environmental effects. Thus, the figure represents a simplified view of how the estimated BVs and RESs reflect the genetic and the environmental components, respectively.

We applied an alternative approach to the estimation of BVs by removing the phenotype of the individual in question from the data file when calculating its BV [24]. Thus, for the 68 males in this experiment, we calculated two types of BVs and environmental residuals (Figure 3). On the one hand the 'traditional' BVs, with the phenotypes of all individuals included in the data file ("BVincl"), and on the other hand, BVs calculated by removing the phenotype of one male at a time from the data file while extracting the BVs ("BVexcl"). The latter was done 68 times for each trait, once for each male. We calculated the reliability of the BVs by dividing the variance in the predicted BVs with the additive genetic variance. As expected, the reliability of the BVexcl was lower than for the BVincl (Figure 3). The correlation between BVincl and BVexcl was high for all three traits: tarsus: r = 0.86, song rate: r = 0.84 and beak colour: r = 0.91.

From the animal models, we extracted the additive genetic variance (V_A_) and the heritability (the ratio of additive genetic variance to total phenotypic variance, [58]) of all traits (Table 1). We also calculated the coefficient of additive genetic variance which standardises the variance with respect to the mean value of the trait (Eq. 2: CV_A_: 100 * V_A_^1/2 ^/trait mean, [59], Table 1).

**Table 1:** Additive genetic variance (V_A_), heritability (h^2^) and coefficient of additive genetic variance (CV_A_) of traits.

Trait	V_A _(SE)	h^2 ^(SE)	CV_A _(%)
Tarsus	0.19 (0.022)	0.55 (0.046)	2.43
Song rate	0.64 (0.22)	0.26 (0.062)	11.9
Beak colour	0.027 (0.005)	0.33 (0.041)	-
Fitness	0.26 (0.19)	0.38 (0.18)	28.5

V_A _of traits is based on tarsus length in mm, the BLUPs of song rate in seconds^1/2^, the PC1 of beak colour, and the relative fitness (number of eggs fertilised/mean number of eggs fertilised). The CV_A _of traits is based on tarsus length in mm, song rate in square-root seconds, and fitness in number of eggs fertilised. Note that CV_A _of beak colour is not computable.

#### Selection analyses using breeding values

We use linear mixed effects models to estimate standardised phenotypic and genetic selection gradients for each of the three traits of interest. By entering several traits simultaneously into the model, it is possible to control for correlated selection acting on traits other than the focal trait [28], to the extent that correlated traits have been measured and can be included in the analysis [60]. Because the genetic, environmental and hence phenotypic correlations between the three focal traits were weak, we chose to use single trait models (additional file 1 Table S1). Table S1 also provides covariances and correlations between the three focal traits and fitness. In general, we aimed to make the models as simple as possible to ease the comparison of the different methodologies. To confirm the validity of our chosen approach, we also performed multivariate analyses and the results were qualitatively the same. An important consideration is the added complexity of a multivariate model which increases the demands on the data. With the current dataset, multivariate models performed less reliably than simpler models. This was especially pronounced in MCMCglmm (see below). The selection gradient for a trait expresses the strength of the linear selection acting on that trait. We focus on linear selection gradients, since our interest is in directional selection on sexually selected traits [27]. Nevertheless, we explored non-linear selection by adding a quadratic term to the linear mixed effect models in a separate analysis. This did not indicate significant stabilising or disruptive selection for any of the three traits and hence was not pursued further (data not shown). We converted male success at fertilising eggs to relative success by dividing the number of eggs a male fertilised with the mean number of eggs fertilised within the aviary. We chose to use aviary mean fertilisation success as the population level mean success because each aviary is in effect a closed population. To obtain meaningful selection gradients, no further transformation of the response is recommended after conversion to relative fitness [28]. In order to make selection gradients comparable across traits, we standardized the trait values prior to the analysis (to mean of zero and SD of one), so that all traits were expressed in units of standard deviations [58]. Standardisation ensures that slope estimates are expressed as changes in units of average fertilisation success per change in units of standard deviations in the predictor variable. The slope of the linear term of the trait represents the (standardised) phenotypic linear selection gradient *β*. Male identity was entered as a random effect, since the observations from the two breeding rounds were treated as separate data points. Models were run once with the phenotypic values of the traits (in order to estimate standardized phenotypic selection gradients), and once with each of the breeding values and residuals (in order to estimate genetic selection gradients standardized by the phenotypic variance of the trait). We use a z-test to evaluate whether two parameter estimates with their standard errors are significantly different from each other. In this way we tested for the phenotypic gambit (genetic and phenotypic selection gradients being of equal strength) and for the equality of genetic versus environmental selection gradients (Figure 1).

#### Selection analyses with a two-trait model

To estimate *β*_A _in one step, we used the MCMCglmm package in R [61] to run animal models with our measure of fitness together with the trait (either tarsus, song rate or beak colour). A Gaussian distribution was specified for all traits. Each model was fitted with one trait and fitness (i.e. relative success within the aviary) as two response variables. To ensure model convergence, we chose bivariate models (rather than multivariate models including all four traits, see above). To simplify calculations, we averaged the relative success from the two seasons to one value per male. To obtain the standardized genetic selection gradients, the genetic covariance between standardized trait and fitness was divided by the additive genetic variance of the trait. MCMCglmm allows sampling from the posterior distribution of the genetic selection gradient (a ratio of covariance and variances) directly. We specified a prior with the variances for all random effects close to 0 (0.002), the covariances between random effects at 0 and the degree of belief parameter (*nu*) set to 2. We ran MCMC-chains for 1000000 iterations (burn-in period: 200000, thinning interval 1000). For comparability, we also fitted all models in VCE 6.0.2, and results were qualitatively the same, although VCE tended to give slightly larger estimates (further from zero).

### Statistics

We used SPSS (SPSS for Windows, Rel. 15.0.1. Chicago: SPSS Inc.) and R 2.9.2 (R Development Core Team, R Foundation for Statistical Computing, Vienna, Austria) for statistical analyses. Mixed model based repeatabilities were calculated following [54] using likelihood ratio tests for testing R = 0 against R > 0. Random effect estimates were obtained with the lmer function (beak colour) and the lme function (song rate). For selection gradient analyses, we used the lmer function from the lme4-package in R 2.9.2 [62]. Animal models were run in REML-VCE 6.0.2 [55] and MCMCglmm (using the MCMCglmm package in R 2.9.2,[61]). All statistical tests are two-tailed.

#### Ethical note

The study was approved by the animal care and ethics representative of the Max Planck Institute for Ornithology.

## Results

**Figure 4:**
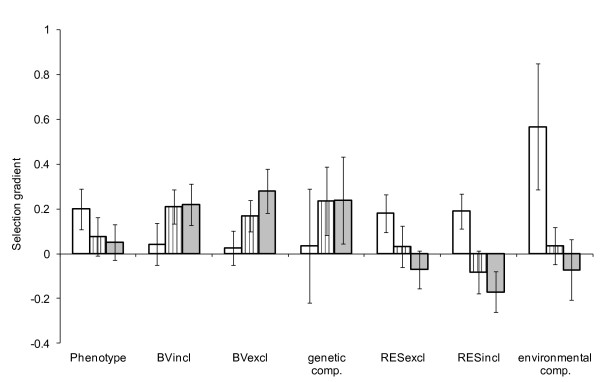
**The relationship between male fitness (number of eggs fertilised under aviary conditions) and each of the three traits: tarsus length (white bars), directed song rate (hatched bars) and beak colour (grey bars)**. The graph compares standardised selection gradients (± SE, standardised to units of phenotypic standard deviations of the trait) calculated using a breeding value approach with genetic selection gradients estimated directly from the covariance between trait and relative fitness in a two-trait model. The different estimations of the genetic and environmental components are ordered from left to right according to the expected bias towards the phenotypic selection gradient. Thus, the phenotypic selection gradient is followed by selection gradients estimated using the traditional breeding value (BVincl), the breeding value calculated with the individual's phenotype excluded from the data file (BVexcl) and finally the selection gradient estimated from the covariance between trait and fitness (genetic comp.). Similarly, the environmental components are ordered from the residual from the phenotype over BVexcl (RESexcl) via the residual from the phenotype over the traditional BV (RESincl) to the environmental covariance between trait and fitness (environmental comp.).

**Figure 5:**
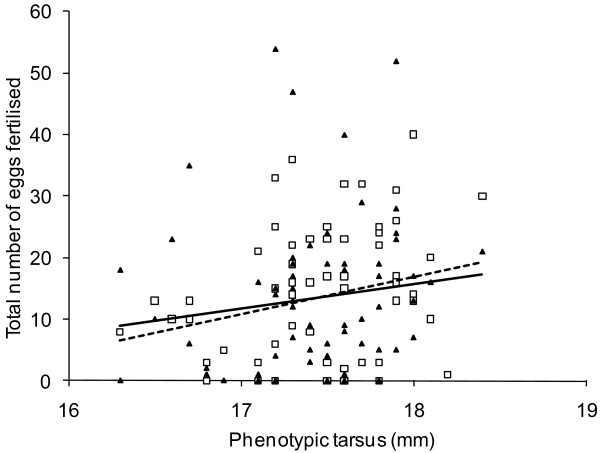
**Phenotypic correlation between tarsus length and male fitness (total number of eggs fertilised)**. Both breeding seasons are included to illustrate the consistency of the effect. Open squares and dashed regression line show season 1, filled triangles and solid regression line show season 2.

At the phenotypic level, standardised linear selection gradients were statistically significantly positive for tarsus (*β*_P _= 0.20 ± 0.09 (SE), Figure 4 and 5), but not for song rate and beak colour (song rate: *β*_P _= 0.076 ± 0.085 (SE), beak colour: *β*_P _= 0.052 ± 0.08 (SE), Figure 4). For all three traits the genetic and environmental selection gradients differed markedly from the phenotypic gradients (Figure 4), yet many of the differences were short of statistical significance (Table 2). The phenotypic selection for longer tarsi was due almost exclusively to an association between the environmental component of tarsus length and the fitness component, while selection at the genetic level was close to zero with all three methods (gradients from two-trait models: *β*_E _= 0.57 ± 0.28 (SE) versus *β*_A _= 0.035 ± 0.25 (SE), Figure 4).

**Table 2:** The significance (determined by z-tests) of the estimates shown in Figure 4.

Trait	Selection gradients for:	P (genetic vs phenotypic)	P (genetic vs environmental)
Tarsus	BVincl	0.23	0.22
	BVexcl	0.14	0.18
	genetic comp.	0.51	0.19
Song rate	BVincl	0.24	0.017
	BVexcl	0.39	0.24
	genetic comp.	0.18	0.27
Beak colour	BVincl	0.17	0.003
	BVexcl	0.08	0.008
	genetic comp.	0.32	0.17

The first column shows the difference between the genetic and the phenotypic selection gradients and the second column the difference between the genetic and the environmental selection gradients.

Interestingly, we found positive, though non-significant, genetic selection gradients for song rate and beak colour, which would suggest evolutionary change (gradients from two-trait models: song rate *β*_A _= 0.24 ± 0.15 (SE), beak colour *β*_A _= 0.24 ± 0.19 (SE), Figure 4). The environmental components of song rate and beak colour showed weak positive or negative associations with the fitness component (song rate *β*_E _= 0.035 ± 0.082 (SE), beak colour *β*_E _= -0.071 ± 0.14 (SE), Figure 4). Comparing the three methods, genetic selection gradients obtained from the two-trait model were generally stronger than those obtained via BVs (Figure 4) but also accompanied by substantially larger standard errors. For example, the difference between the selection gradient for the genetic versus the environmental component of tarsus length was most pronounced with the two-trait model approach. These more extreme genetic selection gradients illustrate how selection gradients are biased towards the phenotype when BVs are used.

## Discussion

Decomposing the phenotypic selection into its genetic and environmental components revealed that the apparent phenotypic linear selection patterns did not reflect genetic patterns for any of the three traits. This suggests that the phenotypes did not directly affect fitness (defined as egg siring success) in a causal way, but rather they were indirectly correlated with fitness because they partly reflect overall genetic or environmental quality of an individual (Figure 1). For tarsus, the linear genetic selection gradient was close to zero while the environmental selection gradient was very strong. This makes it plausible that tarsus length does not respond to selection despite significant linear selection for larger birds at the phenotypic level [see [14] for a similar case in red deer]. Beak colour and song rate, in contrast, showed very weak linear selection at the phenotypic level. The genetic and environmental selection gradients for both song rate and beak colour tended to differ from each other. However, we are cautious in the interpretation of these selection patterns because the standard errors for the genetic and environmental selection gradients were overlapping when using the bivariate approach (i.e. the difference was non-significant, Table 2). Nonetheless, these results might suggest that the phenotypes of these two traits also do not causally affect fitness, that is male-male competition and female choice are not influenced by the phenotypic values of song rate and beak colour. The positive genetic selection gradients would rather suggest that these traits are positively genetically correlated with other unmeasured traits that affect fitness positively. Thus, if the positive genetic covariances with fitness are real, these traits could be seen as reflecting genetic quality to at least some extent [see [63]]. To test the phenotypic gambit, we tested whether the phenotypic and genetic selection gradients were statistically significantly different. Due to a lack of power, this was not the case for any of the three traits (Table 2). This failure to reach significance is unsurprising, given that genetic selection gradients contribute to phenotypic selection gradients, which enhances their similarity.

### Comparing the methods

Compared to the breeding value approach, the two-trait model approach resulted in more extreme genetic and environmental selection gradients that were more different from the phenotypic gradients. This is expected since selection gradients are biased towards the phenotypic gradient when BVs are used, regardless of how the BV is calculated. We expected that selection gradients for the BVincl would more closely reflect the selection at the phenotypic level, while selection gradients for BVexcl should be less biased towards the phenotype and, lastly, genetic selection gradients estimated in a two-trait model should be unbiased with regards to the phenotypic selection pattern (Figure 3). Conversely, selection gradients for the RESexcl should more closely reflect the selection patterns at the phenotypic level compared to the RESincl, while the one-model approach should give an unbiased estimate of the selection gradients for the environmental component.

The observed selection patterns partly correspond to this expectation. However, the differences are mostly minor and some trends are in the opposite direction to the expectation (Figure 4). In general, genetic selection gradients calculated with the use of breeding values showed only a small bias towards the phenotypic selection gradients. This may partly be due to the well connected, error free pedigree available in this study. In studies with less accurate pedigree information (as may often be the case in the wild, especially in populations with high numbers of immigrants) this bias is expected to be more pronounced since less information from relatives is available for each individual to base the breeding value estimation on [see [64]]. The selection gradients at the genetic level of tarsus become progressively less similar to the phenotypic gradients when moving from the BVincl, via the BVexcl to the genetic component. Regarding BVs and their corresponding residuals, this might partly be due to the decreased reliability of the BVexcl (and correspondingly RESincl) since the most informative data point (the individual phenotype) is removed. Thus, the gain from excluding the individual environmental component might in many cases be outweighed by the cost of having less reliable estimates of the genetic merit of an individual. Thus, to exclude the phenotypic value of an individual when calculating its BV may not be an improvement over the use of traditional BVs. However, especially with the use of traditional BVs, caution is required when interpreting selection patterns, since a significant selection on the BVs cannot simply be interpreted as evidence that the trait will respond to selection even in the absence of a genetic constraint. This is also illustrated by a study on antler mass in red deer. Kruuk *et al. *[32] found strong positive selection at the phenotypic level (selection gradient *β*_P_, *= *0.45 ± 0.09 (SE)) of antler mass. With the traditional BV approach, selection at the genetic level was also estimated weakly positive (*β*_A _= 0.16 ± 0.12), suggesting that antler mass should currently be increasing due to positive selection. They also report the genetic correlation between antler mass and fitness (obtained in a two-trait one-model approach) and this was estimated to be negative (r_A _= -0.25 ± 0.29). While the differences in the current study are not as extreme as in this example, it illustrates two important points. First, the importance of moving beyond the phenotype to look at genetic patterns and second, to use a method that captures genetic patterns as far as possible without being biased towards phenotypic patterns.

Hadfield [22] proposed fitting bivariate models that estimate genetic selection gradients directly from one animal model that includes both the focal trait and fitness. This method applied to our data indicated that selection gradients at the genetic and environmental levels, respectively, were somewhat stronger than suggested by the BV method, although this was not vey pronounced with the current dataset. These increased selection gradients were accompanied by larger standard errors. This was expected because the standard errors with the BV approach are downwardly biased [23]. With our dataset, we did not find significant differences between the phenotypic and genetic selection gradients with the bivariate approach (Table 2). This limitation to our study does however illustrate the demands on the data posed by this method. Our data, though based on a 5-generation pedigree of over a thousand birds, is limited by the substantially lower number of individuals with fitness data (68 males). Furthermore, the bivariate approach also revealed significant heritability of fitness in our population. A very low heritability of fitness (which might be the norm when selection pressures are stable, see [65]) would further increase the demands on the data.

### Interpreting the patterns

Considering that the number of fertilised eggs is an important fitness component, its heritability and its coefficient of additive genetic variance were both surprisingly high in our population. Quantitative genetic parameters that are estimated within one generation, (such as fitness in this study) may get slightly inflated estimates of the additive genetic variance due to the inability of the model to accurately separate additive genetic from dominance effects. However, this is likely to have only a minor influence on the heritability estimate and the high heritability in the current study might be due to the substantial change in environmental conditions that the study population was subjected to when entering this study. Current selective pressures will depend on the specific environmental conditions of the experiment. This population of birds has been bred (almost) exclusively in cages for at least 20 generations, with one force-paired pair per cage, thus minimising the opportunity for sexual selection to work. For the current experiment, birds were allowed to breed in free-flying aviaries, allowing for complex social interactions. This drastic environmental change will likely be reflected in current selection pressures, as genes that were selective neutral under cage conditions might become 'good genes' under the new environmental conditions. In other words, male-male competition and female choice will impose selection for genes that are 'good' under the current environmental conditions and these genes will not only reflect 'general good genes' such as for increased health and vigour. In general, the heritability of fitness might be expected to be higher in captivity than in the wild, due to the lower environmental variability in captivity and importantly, the absence of primarily stochastic nest predation.

Male song rate and beak colour were not significantly related to egg siring success. In line with this, previous evidence from this population has shown that females do not prefer males with higher song rates or redder beaks [42]. An apparent preference for the genetic component of these traits could result if females use other cues that are visible on the phenotypic level and genetically correlated with song rate and beak colour (Figure 1). This could be addressed with the use of multivariate models if the relevant traits could be identified. Alternatively, higher song rates and redder beaks may be genetically correlated with egg siring success for reasons other than female choice (there is some evidence that beak colour may be of importance in male-male competition [43,66]).

A positive linear selection on phenotypic body size is common in wild populations [see [67] for a review]. Yet, body size usually remains stable over evolutionary time. This discrepancy may be due to a sampling bias, if selection on body size is measured during periods of intense sexual selection (e.g. the breeding season) and not during period of intense natural selection (e.g. periods of food shortage). The opposing selection pressures would lead to short-term fluctuations in body size in the population while no change in body size would be possible in the long-term since the average food availability does not increase over evolutionary time. Alternatively, it may be the case that an environmental variable (e.g. nutrition during offspring rearing) independently affects both body size and fitness (either directly or through an unmeasured trait). This will lead to a correlation between body size and fitness (see Figure 1). Thus, the observed positive selection on the phenotype may be due entirely to an environmental covariance between body size and fitness [4,68,69]. Then, no change will occur on an evolutionary time-scale (our results are in line with this scenario). Alatalo *et al*. found such a selection pattern on the tarsus length of juvenile collared flycatchers [70]. It is a frequent finding in studies of selection that a trait does not respond to strong and consistent positive linear phenotypic selection for increased trait expression (reviewed in [5]). We would predict that a strong environmental covariance between phenotype and fitness biases the predictions of evolutionary change in a consistent direction. Several studies have addressed this issue but failed to find an environmentally induced bias in selection. This may be due to the use of the BV method, as apparent selection on the genetic level (i.e. on BVs) lead to the rejection of the environmentally induced bias hypothesis [6-8]. We suggest that the apparent paradox of strong selection but no evolutionary response could be addressed through a meta-analysis of studies that have estimated the covariance between e.g. body size and fitness on both the phenotypic as well as the genetic and environmental levels (i.e. not with the BV method). This meta-analysis may reveal that the positive selection on the phenotypic level is due to a positive correlation between the environmental component of the trait and fitness but a low or even negative correlation between the genetic component of the trait and fitness.

## Conclusions

In conclusion, we found a positive phenotypic correlation between fitness (measured as egg siring success) and tarsus, but no selection at the phenotypic level of song rate or beak colour. Decomposing the phenotypic values into the genetic and environmental components revealed a different picture, with a strong environmental, but very low genetic, selection gradient for tarsus, while the genetic components of song rate and beak colour were positively associated with fitness. This illustrates the importance of separating the genetic and environmental components of traits. This is best done through the application of models that directly estimate the covariance between trait and fitness in a bivariate animal model. This provides a maximally efficient method to decompose the phenotype into its genetic and environmental components and allows estimation of selection parameters with realistic error estimates. Yet, in this study, we could not reject the null hypothesis that genetic and environmental selection gradients are identical. This highlights the power issues with this approach, which requires large sample sizes, especially if the heritability of fitness is low. In general, a phenotypic perspective is not enough to elucidate the causal relationship between a trait and fitness because phenotypic selection gradients can give a misleading picture of evolutionary change.

## Authors' contributions

EB helped to conceive of and perform the study, performed the statistical analyses and wrote the manuscript. HS helped to conceive of and perform the study, and assisted with the statistical analyses and with writing the manuscript. WF conceived of the study, and participated in its design and coordination and helped writing the manuscript. All authors read and approved the final manuscript.

## Supplementary Material

Additional file 1 **Genetic (_A_) and environmental (_E_) covariances (cov) and correlations (r) between song rate (BLUPs of song rate, in seconds^1/2^), beak colour (PC1), tarsus length (mm) and fitness (number of eggs fertilised/mean number of eggs fertilised). Cov_A _and cov_E _adds up to the phenotypic covariance, and likewise for the correlations**. Click here for file
