# Supplementary material for: Correlates of male fitness in captive zebra finches - a comparison of methods to disentangle genetic and environmental effects
Source: BMC Evol Biol. 2011 Nov 8;11:327. doi: 10.1186/1471-2148-11-327 (PMC3229482; doi:10.1186/1471-2148-11-327)
Supplement: Additional file 1 — Genetic (A) and environmental (E) covariances (cov) and correlations (r) between song rate (BLUPs of song rate, in seconds1/2), beak colour (PC1), tarsus length (mm) and fitness (number of eggs fertilised/mean number of eggs fertilised). CovA and covE adds up to the phenotypic covariance, and likewise for the correlations. [file 1471-2148-11-327-S1.DOCX]

Additional file 1, Table S1.

Genetic (_A_) and environmental (_E_) covariances (cov) and correlations (r) between song rate (BLUPs of song rate, in seconds^1/2^), beak colour (PC1), tarsus length (mm) and fitness (number of eggs fertilised/mean number of eggs fertilised). Cov_A_ and cov_E_ adds up to the phenotypic covariance, and likewise for the correlations.

|  | cov_A_ (SE) | cov_E_ (SE) | r_A_ (SE) | r_E_ (SE) |
| --- | --- | --- | --- | --- |
| Song-tarsus | -0.0077 (0.041) | 0.046 (0.030) | -0.022 (0.12) | 0.11 (0.075) |
| Tarsus-beak colour | -0.0094 (0.0064) | 0.015 (0.004) | -0.13 (0.089) | 0.20 (0.054) |
| Song-beak colour | -0.014 (0.017) | 0.043 (0.016) | -0.11 (0.13) | 0.17 (0.064) |
| Song-fitness | 0.25 (0.12) | 0.037 (0.096) | 0.64 (0.25) | 0.05 (0.13) |
| Tarsus-fitness | -0.036 (0.040) | 0.15 (0.034) | -0.16 (0.17) | 0.67 (0.16) |
| Beak colour-fitness | 0.040 (0.013) | -0.013 (0.014) | 0.50 (0.21) | -0.10 (0.10) |
